# Laser-Assisted Depigmentation—An Introspection of the Science, Techniques, and Perceptions

**DOI:** 10.3390/dj8030088

**Published:** 2020-08-06

**Authors:** Alex Mathews Muruppel, B.S. Jagadish Pai, Subraya Bhat, Steven Parker, Edward Lynch

**Affiliations:** 1 Department of Surgical Sciences and Integrated Diagnostics, University of Genoa, 16132 Genova, Italy; 2 Faculty for Diploma in Laser Dentistry, University of Genova in India, Bangalore 560071, India; drjagadish.pai@apoorvahospital.com; 3 College of Dentistry, Imam Abdulrahman bin Faisal University, Dammam 32241, Saudi Arabia; sgbhat@iau.edu.sa; 4 Leicester School of Pharmacy, De Montfort University, Leicester LE1 9BH, UK; thewholetooth@easynet.co.uk (S.P.); edward.lynch@dmu.ac.uk (E.L.); 5 School of Dental Medicine, University of Nevada Las Vegas, Las Vegas, NV 89154, USA

**Keywords:** depigmentation, gingival hyperpigmentation, lasers, melanin

## Abstract

Background: Gingival hyperpigmentation is a major concern for a significant number of patients, as a relevant aesthetic or cosmetic need. Oral melanin pigmentation is considered to be multifactorial and could be related to physiological or even pathological reasons and can be the consequence of a variety of local or systemic factors. This pigmentation varies individually across races or age groups and is without any gender predilection. Evidence gleaned from literature is presented from case–control studies and from the authors’ own research work in prospective, split-mouth, double-blinded, clinical trials comparing treatment modalities in effecting depigmentation. Methods: A systematic review of published articles, using suitable assay criteria, was carried out to formulate a consensus on laser-assisted modalities. A total of 295 published sources were subject to critical analysis and resulted in six papers that were subject to data scrutiny. Additionally, evidence is presented on clinical protocols and treatment outcomes. Results: Analysis of randomized clinical studies identified the use of two laser wavelength groups—near infrared diode and erbium group of mid-infrared lasers. Several areas of analysis were examined, and inconsistent degrees of significance were obtained to establish which laser group was optimal and if they were any better than scalpel depigmentation. Conclusion: A definitive conclusion is wanting as studies with scientific and standardized protocols of evaluation are yet to provide a take on comparative assessments between different techniques of depigmentation.

## 1. Introduction

The color of the gingiva depends on multiple factors of race, systemic and local, physiologic, and even pathologic factors and could range from pale pink to coral pink, to deep red, and even violet depending on the pigmentation of the gingiva by five primary pigments. These include melanin, melanoid, oxyhemoglobin, reduced hemoglobin, and carotene [1], of which melanin pigmentation is the most common [1]. Systemic endocrine imbalances or pharmacotherapeutic agents and even exogenous agents such as heavy metals (mercury, bismuth, lead, iron) can predispose to gingival hyperpigmentation [2,3,4]. Hirschfeld and Hirschfeld stated that oral pigmentation was often considered as a manifestation of Addison’s disease at that time and termed it as “melanoplakia” [5].

Melanin, a non-hemoglobin derived brown pigment, the most common of the endogenous pigments is produced by the specialized cells called melanocytes situated in the basal layer of the oral epithelium but is transported through melanosomes (an ovoid granule-like organelle, 400–800 nm) into superficial layers of keratinocytes in the stratum spinosum, which is only 0.027–0.15 mm thick [6]. The superficial stratum corneum of 0.01–0.02 mm, consisting of dead and exfoliating cells [5], does not hold significance in our context of pigmentation, other than in its ability to scatter light. This pigmentation is symmetric and persistent, and it does not alter the normal architecture of the epithelium [7].

As such, gingival hyperpigmentation in itself does not present a problem, but especially when associated with a gummy smile, patients complain of “black gums” and request cosmetic correction [8]. Wide-ranging variation in gingival pigmentation in people of different races occurs as a result of the relative activity of the melanocytes in producing melanin, due to the size of the melanosomes (which are larger in Negroid subjects and smaller in Caucasian or other races), and also from the rate at which melanosomes are broken down and dispersed into the keratinocytes.

Various therapeutic modalities have been employed for depigmentation of the gingiva, including gingivectomy, gingivectomy with free gingival auto grafting, electrosurgery, cryosurgery, and different laser wavelengths too, and this article hopes to present a scientific assessment of various methods for effecting gingival depigmentation.

The literature reports various classifications of the techniques employed for gingival depigmentation techniques from the earliest classification by Dummett et al. in 1945 from Class I–Class IV [2] (Table 1 and Table 2) [9].

A further classification is offered in the Peeran et al. (2014) published paper and is represented as follows, using a score of 0–10 with associated pathology. Table 3 [10,11].

Furthermore, in the Jepsen et al. publication (2018) [11], there is a single reference to gingival color/pigmentation with referenced pigmentation within Mucogingival Deformities and Conditions Around Teeth under #3(g).

A review of published data subject to strict selection criteria was undertaken, to evaluate the parameters by which laser-assisted depigmentation treatment may be safely delivered and to examine underlying factors relating to etiology and therapy options.

## 2. Materials and Methods

### 2.1. Search Strategy

An electronic search was conducted relating to laser-assisted gingival depigmentation applications in all fields of dentistry from 17 to 21 July 2020. Databases used were PubMed, Cochrane, and Google Scholar, with the following MeSH terms, keywords, and their combinations: Laser AND (oral or gingiva) AND (pigmentation OR depigmentation).

**Figure 1:**
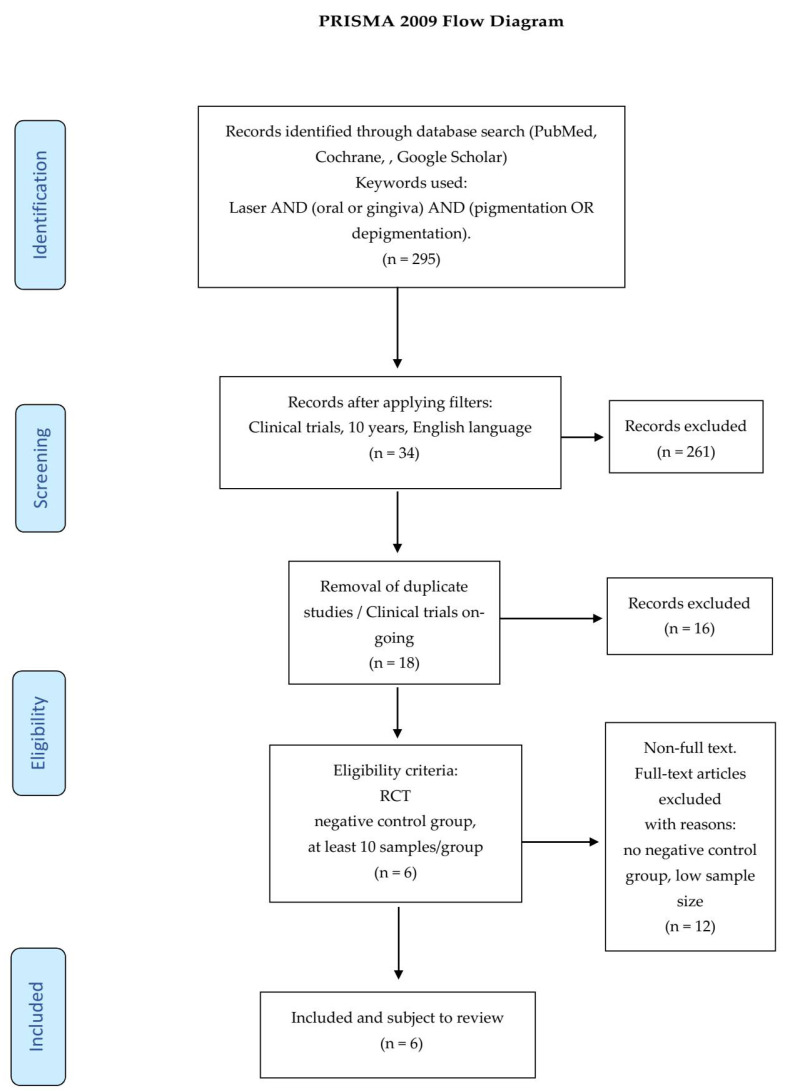
Preferred Reporting Items for Systematic Reviews and Meta-Analyses (PRISMA) flow-chart of selected criteria for the included article reports [12].

After applying the additional filters referenced in the Preferred Reporting Items for Systematic Reviews and Meta-Analyses (PRISMA) statement (Figure 1), the preliminary 295 articles were reduced to 6.

Titles and abstracts of the above articles were independently screened by two reviewers via application of the following criteria. In case of any disagreements arising, these were satisfactorily resolved by discussions.

Inclusion criteria

- laser used as light source,
- at least 10 samples/patients per group,
- only randomized clinical trials and studies,
- a minimum of a 3 month follow-up.

Exclusion criteria

- duplicates or studies with the same ethical approval number,
- no negative control group,
- low sample/patient sizes (less than 10 per group),
- no randomized controlled clinical trials or pilot studies,
- <3 month follow-up.

After screening and implementation of the eligibility criteria, a total of 6 articles were retained.

In accordance with the PRISMA statement [12] details of the selection criteria are presented in Figure 1.

### 2.2. Data Extraction

Having reached a consensus regarding the selection of included articles, the two reviewers involved subsequently extracted data regarding:

- citation (first author and publication year),
- type of study/number of samples,
- test/control groups,
- follow-up,
- outcome.

### 2.3. Quality Assessment

Subsequent to data extraction, articles were further evaluated by assessing their risk of bias. The Cochrane Risk of Bias tool [13] was modified according to the requirements of this systematic review.

The risk of bias was determined according to the number of “yes” or “no” responses to the parameters provided below, which were allocated to each study.

- randomization,
- sample size calculation and required sample numbers included,
- baseline situation similar to that of the test group,
- blinding,
- parameters of laser use described appropriately and associated calculations correct,
- power meter used,
- numerical results available (statistics),
- no missing outcome data,
- all samples/patients completed the follow-up evaluation,
- correct interpretation of data acquired.

The classification was performed according to the total number of “yes” answers to the above questions. For the current study, the degree of bias was computed according to the score limits provided below:

High risk: 0–4,
Moderate risk: 5–7,
Low risk: 8–10.

## 3. Results

A detailed description of the complete set of data extractions and parameters applied, plus a digest of associated information was compiled. As noted above, 6 low-risk papers fulfilled the defined limits of this systematic review in Table 4.

## 4. Discussion

A systematic review of published data has examined the optimal laser-assisted treatment modalities. Such therapy may be considered among the many alternatives as follows.

### 4.1. Classification of Gingival Depigmentation Techniques

#### 4.1.1. Topical Application (Chemical Cauterization)

In the earliest reported paper on gingival and skin pigmentation and its classification, Hirschfeld and Hirschfeld [5] described a method of depigmentation applying 90% phenol “for an instant” and thereafter following up with 95% alcohol (both were applied using cotton wool) to “neutralize” the effect of the phenol. This regimen was repeated until the area of pigmentation was covered comprehensively. The scab formed by such cauterization would eventually fall off and the area heals in 10–14 days. Yamamoto et al. [20] reported a definite inflammation and destruction of vascular endothelial cells in the dermis more than actual keratinocytes of the lower epithelium after such treatment with phenol.

**Figure 2:**
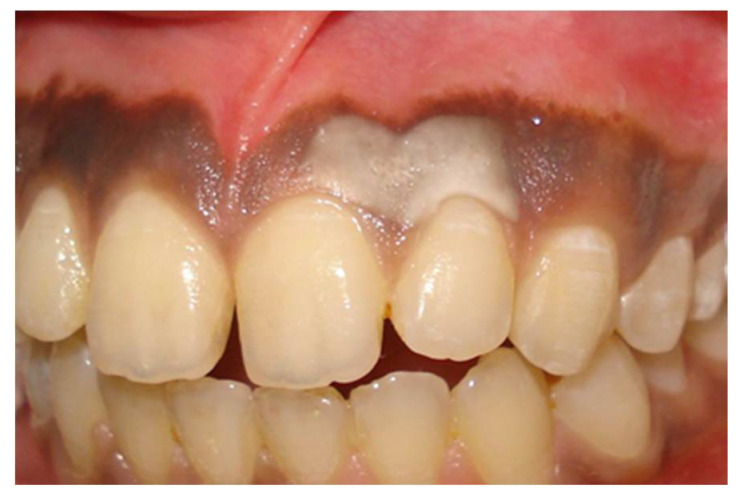
Tissue blanching seen immediately after application of tetrafluoroethylene (TFE) due to rapid freezing of tissue (courtesy Dr. S. Bhat).

**Figure 3:**
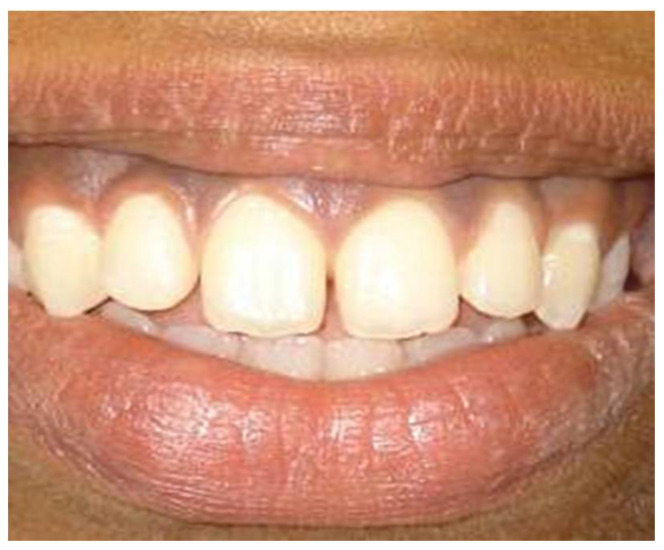
Pre-operative before depigmentation with tetrafluoroethylene (TFE) (courtesy Dr. S. Bhat).

**Figure 4:**
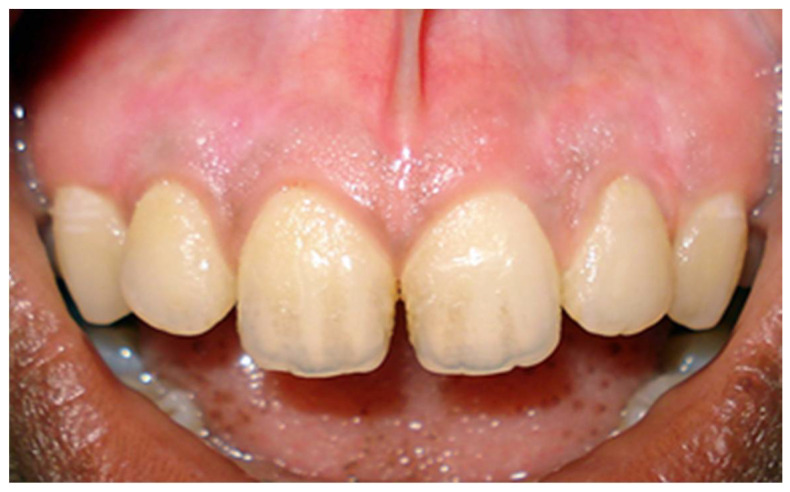
Post-operative follow-up after 180 days—TFE depigmentation (courtesy Dr. S. Bhat).

The pioneering report by the Hirschfelds became the basis of the use of other agents such as tetrafluoroethane (a common refrigerant) and even liquid nitrogen in a similar manner for depigmentation. Only the process of application differed when using a cryoprobe or gun and the basis of the tissue destruction here is by rapid freezing of tissues (Figure 2, Figure 3 and Figure 4).

Tal et al. [21] reported that the patient had pain during this procedure, yet it could be done without local anesthesia and complete healing occurred in 3–4 weeks with no relapse noted even after 20 months. Yeh [22] practiced the same technique on 20 patients albeit applying liquid nitrogen with a cotton swab without the help of local anesthesia and though there was erythema and inflammation [23] for the next 3 days, the gingiva appeared normal by 4 weeks and no relapse of pigmentation was reported even after 2 years [24].

**Figure 5:**
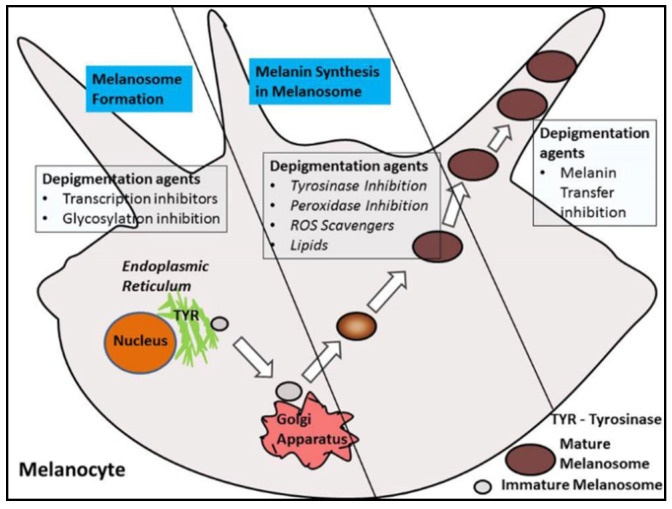
Topical depigmentation agents and their mode of action.

The other topical agents employed in depigmentation interfere with or inhibit the production of melanin. Ascorbic acid checks dopaquinone [25] formation (a precursor to melanin), similarly, other agents act by regulating (a) the formation and activity of the enzyme tyrosinase and its related family of enzymes (tyrosinase related protein-1 (TRP-1) and tyrosinase related protein-2 (TRP-2)), (b) the reception and diffusion of melanosomes in keratinocytes, and (c) actual maturation of pigmented keratinocytes (Figure 5).

**Figure 6:**
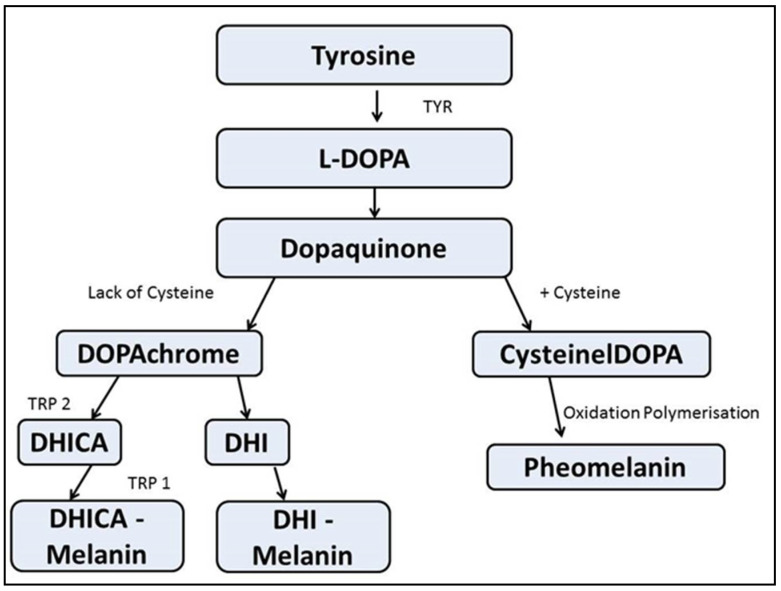
Cascade of melanin synthesis in melanocyte.

Shimada et al. [26] analyzed the amount of melanin formed using a spectrophotometer in the in vitro leg of the study. In the in vivo part of the study, a double-blinded placebo-controlled trial was conducted on 73 volunteers between 25 and 57 years of age, where the ascorbic acid gel was applied once daily every night for 12 weeks and evaluated every 4 weeks. The results showed that ascorbic acid as a depigmentation agent did reduce melanin formation in the cultured cells, but no significant change was observed in the 3D skin model; however, in all the volunteers, there was a significant lightening of the gingiva. This initial study proved the effect of ascorbic acid 2-glucoside on inhibition of the enzyme tyrosinase (Figure 6).

#### 4.1.2. Surgical Techniques

Ginwalla et al. [27] suggested an abrasive surgical technique in which depigmentation was carried out with burs. However, this technique is time consuming, is painful post-operatively, and 80% of the cases treated showed re-pigmentation at the end of six months [28]. Almas et al. (2002) [29] treated a 28-year-old male Caucasian on both maxillary and mandibular arches, using a simple surgical approach with a scalpel blade, initially pioneered by Dummett and Bolden in 1963 [30], and the patient was monitored for a longitudinal period for any reappearance of pigmentation in the follow-up period of 6 months, and there was no relapse of pigmentation within this duration of follow-up. They concluded that the technique was very simple as it did not require sophisticated or expensive armamentarium.

Perlmutter and Tal (1986) [31] demonstrated the removal of pigmented keratinized gingiva by surgical scalpel gingivectomy in two Jewish Yemenite adult males. After surgery, the exposed connective tissue was covered by a periodontal pack for 7–10 days. The tissues were then observed periodically for signs of repigmentation. They reported that the healing was uneventful in both patients; however, 32 months later, the pigmentation returned and subsequently the area was completely repigmented after 7 years in the first patient.

Free gingival grafts are used to create a widened zone of attached gingiva. They were initially described by Bjorn [32] and have been extensively investigated [33,34]. Free gingival grafts can be used effectively for the treatment of gingival hyperpigmentation. The disadvantage was the color mismatch between the recipient and donor tissues (mostly the graft is procured from the palate).

**Figure 7:**
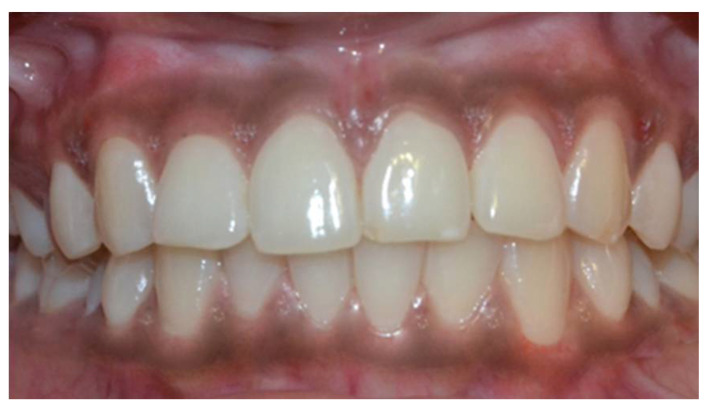
Pre-operative—comparison between scalpel, cryosurgery, diode laser 810 nm study (Pai et al.).

**Figure 8:**
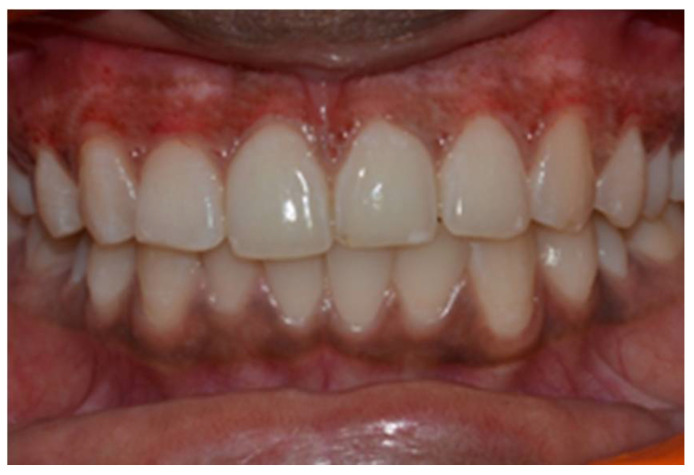
Immediate post-operative—depigmentation by diode laser 810 nm on upper arch (Pai et al.).

**Figure 9:**
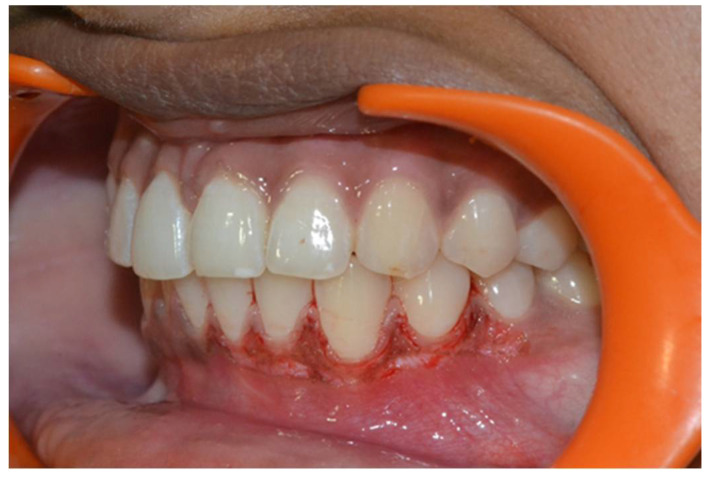
Immediate post-operative—depigmentation by scalpel on lower arch left quadrant (Pai et al.).

**Figure 10:**
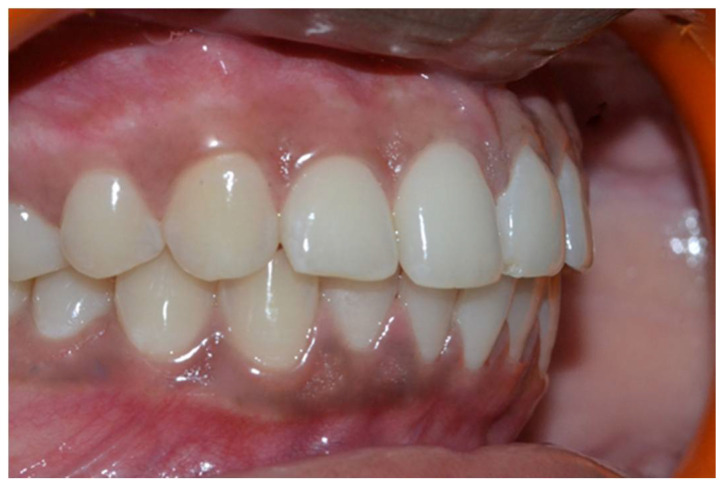
Immediate post-operative—depigmentation by cryosurgery on lower arch right quadrant (Pai et al.).

**Figure 11:**
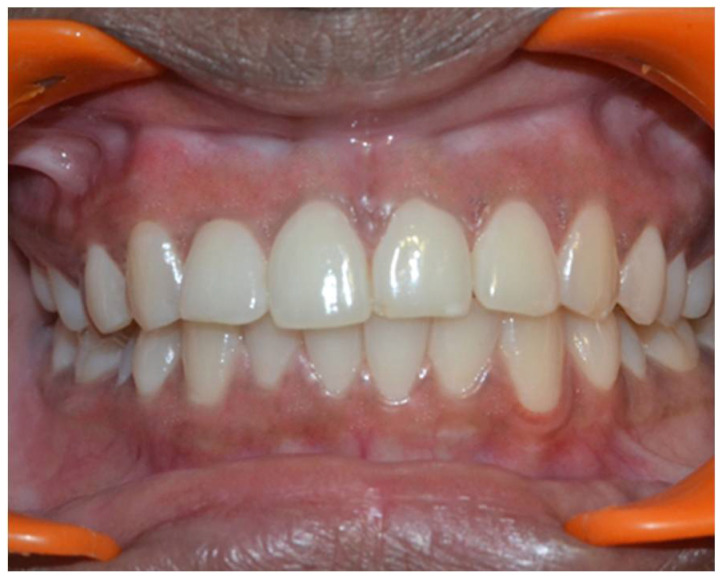
Post-operative follow-up after 1 year—comparison between scalpel, cryosurgery, diode laser 810 nm study (Pai et al.).

In a prospective, split-mouth, double-blinded trial (Pai et al.), to compare and evaluate the effectiveness of cryosurgery, diode laser 810 nm, and conventional surgical procedure using scalpel in the management of gingival hyperpigmentation. Laser ablation and cryosurgical procedures were found to be superior to conventional scalpel technique in terms of intra-operative bleeding and post-operative pain, whereas wound healing was complete and uneventful within one week without any post-operative complications (Figure 7, Figure 8, Figure 9, Figure 10 and Figure 11).

Though surgical approaches, using either the abrasive techniques with burs or scalpel are inexpensive [35], they need to be done under effective local anesthesia, pressure needs to be applied to achieve hemostasis and this increases patient discomfort intra-operatively [36]; this technique also mandates the use of a periodontal pack as a surgical dressing post operatively. The healing of the denuded surgical area where the connective tissue is now devoid of epithelium is by secondary intention and therefore has a period of associated morbidity; convalescence of 7–14 days is advised, wherein the patient may not be able to enjoy normal diet. There also is a higher incidence of relapse because of melanocyte shift from the surrounding areas [37].

#### 4.1.3. Electrosurgery

Electrosurgery facilitates easy tissue incision accompanied with a strong hemostatic effect. However, the thermal effect of electrosurgery is relatively stronger, and the major concern is the potential risk of thermal damage to the underlying periosteum and alveolar bone by direct contact of the electrosurgical tip during gingival tissue management, thereby leading to necrosis of bone or causing delayed wound healing [38]. Depigmentation through electrosurgery works on the principle of fulguration or electrosurgical fulguration (arcing between the electrode tip and the conductive target tissue inducing coagulation in tissue), which coagulates and could even char the tissue over a wide area [39].

The end result here is the creation of a coagulum or charring rather than actual cellular vaporization. The resultant thermal effects and collateral damage (edema from coagulative necrosis) could cause a measure of post-operative pain and discomfort to the patient that would be proportional to the voltage used and the exposure time to the tissue, which in turn also prolongs the healing period [40].

Gufran (2016) [41] compared depigmentation with scalpel and electrosurgery. The loop electrode was used, in combination with a fully rectified current, in the intention of cutting smoothly and also providing coagulation. The clinical findings showed slight redness around the margins of the surgical site as soon as the electrode was removed, and coagulation was also effective. They observed that thereafter, a thin film formed at the coagulated site as the site healed, which eventually disappeared. They covered the wound with a periodontal pack for 7 to 10 days and found after 7 days that epithelization was completed. They found that there was no significant difference between both groups in terms of the pigmentation as assessed by the Dummett—Gupta Oral Pigmentation Index (DOPI) [2] even after 6 months, though they suggested that the scalpel technique was simpler and effective.

These findings were consistent with Prasad et al. (2005) [42] in whose case a series of bur abrasion, partial thickness flap (epithelial excision) using no. 15 scalpel, cryotherapy, and electrosurgery had been employed for depigmentation, but the abrasive surgical technique and scalpel technique showed a relapse of pigmentation after 3 months.

The findings also concur with Sherman (2009) [43] in which depigmentation was done by touching the pigmented areas lightly with the no. 135 ball-shaped electrode or tapping the area with the no. 134 L-shaped electrode. The technique showed good results, and the epithelization was completed in 10 days.

#### 4.1.4. Lasers

Melanin performs essential, indispensable, and integral functions in the skin such as providing photoprotection against harmful UV rays, as an antioxidant, and even possessing drug binding properties, as in relation to nicotine in smokers [8,44]. However, most notable, in our context of depigmentation, is the fact that lasers are an effective, minimally invasive, and often painless alternative to the conventional and traditional techniques of depigmentation. Lasers of a range of wavelengths in dentistry, from the visible spectrum such as the “blue” and “green” diode lasers (440, 532 nm), near infrared diode lasers (810, 940, 980 nm), mid-infrared lasers Nd:YAG (1064 nm), have a broad affinity for pigmented chromophores, including melanin. Mid-infrared erbium YAG (2940 nm) and erbium chromium YSGG (2780 nm) and far infrared CO_2_ lasers (10,600 nm) have high absorption in water; as such are all effective in performing depigmentation. However, the mechanism differs in that the shorter visible and near infra-red wavelengths will pass through the surface layer of cells to target the basal membrane and melanin deposits and denature the structure. Conversely, the longer wavelengths may perform an action similar to a “laser peel”, whereby the surface layer by layer is vaporized until the melanin layer is finally ablated. In both procedures, care should be exercised to effect depigmentation but avoid deeper risk of damage, including thermal conduction through to the underlying bone and/or dental root and pulp. One of the reasons for this is the broadband absorption spectrum of melanin as a molecule and the fact that the melanosomes into which it is packaged are rich in protein and water (up to 93%) [45,46]. This absorption of the laser light’s energy means that practically one does not have to de-epithelize the entire pigmented area but can be conservative and allow the light to degranulate the melanosomes (by protein absorption) and or denature the melanin. Denatured or degranulated melanin is the reason why after laser-assisted depigmentation the post-surgical area appears light brownish. The surgical area then heals following a definite sequence of tissue events wherein the degranulated melanin disappears.

Laser emission mode may affect the thermal relaxation capacity of the treatment area; continuous wave emissions often seen with visible, near IR diode, and CO_2_ wavelengths should be used with caution to avoid over-heating. Additionally, the deeper tissue penetration and free-running pulsed delivery of the Nd:YAG may pose some risk of high peak power values. Overall, a detailed appreciation of using the minimum amount of power sufficient to achieve a desired result should be employed.

##### Contact Mode

In one of the earliest reports of the use of lasers for depigmentation, Trelles et al. (1993) [47] used the argon laser (514 nm) at 1.5 watts (W) of power in pulsed mode of 300 milliseconds, 50% duty cycle, and a spot size of 0.5 mm using a fiber tip, in contact mode and under local anesthesia in 36 patients. Most patients (except 2) did not require any post-operative analgesic and the depigmentation result was good in all 34, except in two patients who required a second session of treatment. However, there was no mention of the follow-up period or relapse of pigmentation.

Atsawasuwan et al. (2000) [48] described a case report detailing the use of the Nd:YAG laser for depigmentation at 6 W in pulsed mode at a frequency (Hz) of 100 Hz, 6 mJ per pulse, 320 µm fiber tip in contact mode in 4 patients. The healing was uneventful and there was no relapse of pigmentation in 11–13 months of follow-up. There is no mention of any local anesthetic having been given either as a topical application or as an injection, but none of the patients required any analgesic post-operatively though.

Ribeiro et al. (2012) [18] assessed the effectiveness of depigmentation done using the scalpel technique as compared to the Nd:YAG laser in a randomized clinical trial. One side of the anterior maxillary arch was randomly assigned for treatment with either modality. They assessed patient responses to pain on the Visual Analog Scale (VAS) and also graded the reappearance of pigmentation from poor to excellent on a predecided arbitrary scale of 5 grades. They reported that the scalpel technique took longer, and the patient felt more pain post-operatively as compared to the Nd:YAG laser. After 6 months, the patients’ satisfaction in terms of esthetics was similar though between scalpel and Nd:YAG laser.

Chandra et al. (2020) [15] in a randomized clinical trial with a split-mouth design, compared and assessed depigmentation done with scalpel technique and diode laser (810 nm)-assisted depigmentation for bleeding, pain in the post-operative period on a VAS, and also the recurrence of pigmentation after 6 months using the DOPI. They found that the side treated with the scalpel technique healed faster, but patients experienced more bleeding during and immediately after the procedure. However, both pain (after 1 day and 1 week) and recurrence of pigmentation (after 6 months) were comparable between both sides though the diode laser side had slightly lesser percentages of pain and recurrence of pigmentation, which was not statistically significant.

Basha et al. (2015) [14] compared depigmentation done using the Nd:YAG laser at 3 W, 30 mJ per pulse, in contact mode, using a 300 µm fiber tip, under topical anesthetic only, on one quadrant of the maxillary arch, in contrast to surgical stripping of the pigmented gingiva done with a no. 15 scalpel under local anesthetic injection on the contralateral quadrant of the maxillary arch in 20 patients using a split-mouth approach. They found that the time taken for the procedure was much less and, furthermore, a bloodless and clear surgical site was obtained on the laser side; also in terms of patient preference, it was far less painful, a majority of patients preferring the laser as the more comfortable treatment modality. However, the assessment of repigmentation at 6 months showed the laser side having a slightly higher incidence of repigmentation as opposed to the surgical side, though the difference was not statistically different.

Chandna et al. (2015) [49] showed through their study comparing pain levels between electrosurgery and diode lasers that at all intervals (24 h, 1 week) the patients in the diode lasers group experienced significantly lesser pain levels as evaluated by the Visual Analog Scale (VAS).

Raaman et al. (2017) [50] studied the post-operative pain on 1st, 4th, and 7th days as evinced by the VAS and compared repigmentation rates on the 30th and 90th days in 25 patients each treated with scalpel and the 980 nm diode laser. They found that all patients reported a painless experience with the lasers, while repigmentation rates where similar between both groups of patients treated by both modalities.

Nd:YAG and diode lasers (both visible and near infrared) have optic fiber energy delivery and therefore permit depigmentation in contact mode. While this gives the operator some tactile feedback, the procedure needs to be done in light, broad, sweeping brush strokes of the fiber tip in a feather touch, moving in an apicocervical direction (from the attached gingiva downward to the marginal gingiva). Care should be taken not to dwell on any one area for a long time as this can cause an exposure time that may outweigh the thermal relaxation time of the tissue and will cause deeper collateral damage and delayed healing. Care should be taken not to nick the thin marginal gingiva too as this can mean unpredictable healing with rolled gingival margins and even cause slight gingival recession. All the three lasers described above also give the distinct and minimally invasive advantage that they allow selective ablation of melanin, melanosomes, and melanocytes because of their high direct absorption in melanin and excellent hemostasis due their high absorption in hemoglobin [51]. Luk and Anagnostaki (2017) reported the use of diode lasers of the visible spectrum of 455 nm (blue), for a “non-ablative” depigmentation technique. At this wavelength, the peak absorption of melanin and hemoglobin combined allows exceptionally effective and selective depigmentation by a rapid local increase of temperature in the tissue (photocoagulation) [52].

##### Non-Contact Mode

**Figure 12:**
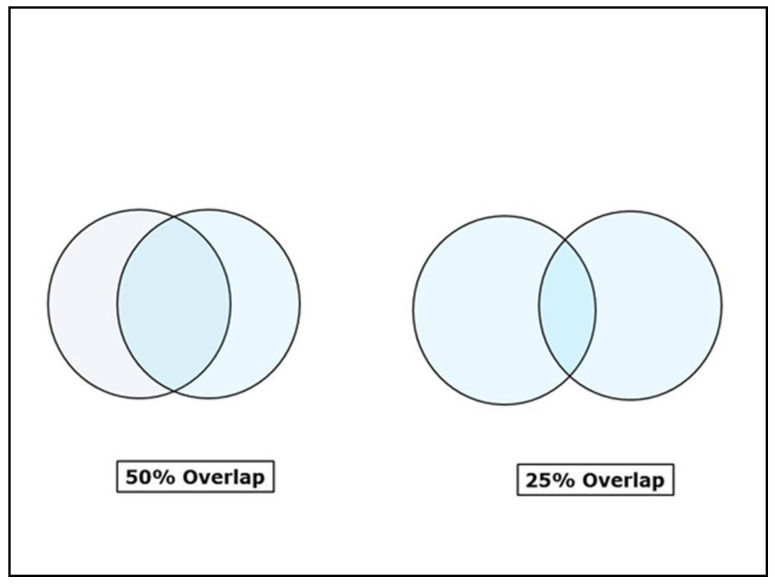
Laser spot overlap—depigmentation with pulsed lasers such as Er:YAG, Er,Cr:YSGG, and CO_2_ (in pulsed mode) should have at least 25–50% overlap between pulses.

The erbium family of lasers (Er:YAG and Er,Cr:YSGG), CO_2_ lasers, and the newer generation superpulsed diode lasers (using a glass rod instead of a fiber tip) allow the operator to work in non-contact mode. This technique gives a great deal of comfort to the patient as it obviates the scraping feel of the fiber tip during the procedure. Working in non-contact mode requires a great deal of precision and accuracy. With the erbium lasers, depigmentation is achieved with minimal water spray, and the laser delivers energy in free running pulsed mode through sapphire or zirconia tips and the pulses show as opaque spots on the gingiva. These spots need to have a minimum of 20–30% overlap [53] and ideally around 50% overlap (Figure 12) otherwise, the space between two succeeding spots would remain untouched and the pigmentation would show up in these areas on healing.

As one progresses, there would be flakes of desiccated and ablated epithelial tissue, which would fly up as if it was brushed up and hence this technique of depigmentation using erbium lasers is termed as “brush technique”. Once an area of the gingiva has been covered, a good practice would be to either wipe away the desiccated epithelial layer with moist gauze or remove it with the convex outer bend of a curved explorer and then revisit the areas that still show pigmentation. With deeper progress there would be some bleeding. The erbium lasers are very gentle, having a penetration of only 2–4 µm in the case of the Er:YAG, and only around 15 µm in the case of the Er,Cr:YSGG lasers [54], so one must work layer through layer until the area is free from pigmentation. The target chromophore or primary absorber of the laser energy here is water, and then the protein of the epithelium and melanosomes, but notably no absorption in hemoglobin would translate as poor hemostasis clinically.

Tal et al. (2003) [55] used the Er:YAG laser at 500 mJ pulses, at a frequency of 10 pulses/second, and spot size of 3 mm using a free running beam (FRB or tipless handpiece) from a distance of 7 mm away from the tissue on 10 patients who needed depigmentation under only topical application of 2.5% lidocaine. The patients were asked to fill out a Melzack’s McGill pain questionnaire. They were evaluated at 1, 2, 3, and 6 months for reappearance of any pigmentation. Most of the patients described the treatment procedure as pleasant, and only 3 patients used analgesics post-operatively. There was no reappearance in pigmentation in any of the patients even after 6 months.

Rosa et al. (2007) [56] evaluated the depigmentation performed on 5 patients using an Er:YAG laser, with 64 mJ/pulse, at a frequency of 10 Hz with water spray using a 0.5 mm tip under topical and local anesthesia injection. They evaluated the post-operative pain using a VAS and reappearance of pigmentation was evaluated at 1 and 3 months. They reported 3 patients did complain of moderate pain, but there was no reappearance of any gingival pigmentation during the time of follow-up.

When working with CO_2_ lasers, one would not have the relative control of delivering the energy through tips as in erbium lasers, and the articulating arms that deliver the energy in CO_2_ lasers are a bit bulky and cumbersome. It takes a learning curve to focus a spot size of say, 0.8 mm from a distance of 1–2 cm away.

The CO_2_ laser is effective and particularly well suited for treating gingival hyperpigmentation, as its wavelength (10,600 nm) is highly absorbed by the water content of soft tissues. This high absorption entails that it has minimal penetration (around 50 µm) and, therefore, would not pose a danger to the periosteum and bone under the gingiva being treated. It also has the unique characteristic of being able to remove a thin layer of epithelium by inducing a blister formation and, thereafter, detaching the entire epithelium perfectly. This has been described as the “epithelial peel technique” by Hegde et al. (2013) [57]. The CO_2_ laser performs elective ablation of melanocytes by removing epithelial cells judiciously to the basal layer of the treated area.

Ozbayrak et al. (2000) [58] used the CO_2_ laser between 5 and 7 W, in a pulsed mode on the gingiva, labial mucosa, and alveolar mucosa for treating constitutive pigmentation (skin pigmentation that occurs by genetic predisposition) and facultative smoker’s melanosis (hyperpigmentation occurring due to exposure to UV light, nicotine, hormonal, or factors other than genetic predisposition) under local anesthesia in a total cohort of 8 patients. Re-epithelization following the procedure occurred within 2 to 3 weeks with minimum discomfort, although 2 individuals had pain post-operatively. Ablation of the hyperpigmented gingiva was accomplished with minimal bleeding. The patients were evaluated every 3 months over 18 months, and there was no relapse of the pigmentation.

Hedin et al. (1977 and 1992) [59] described the occurrence of facultative pigmentation in smokers occurring intra-orally in the anterior gingiva of the maxilla and mandible even in people of fair-skinned races. They concluded from their studies that smoking would further stimulate the melanocytes to induce more pigmentation [60]. They attributed this to Lindquist and Ullberg’s (1975) surmise that nicotine’s affinity to melanin [61], similar to that in the hair and inner ear, might serve as an answer to this phenomenon [62,63].

Nakamura et al. had previously demonstrated, first in 5 dogs (1992) [64], and thereafter (1999) [65] in clinical treatments, and through histopathological evaluations, on 10 patients (of which 7 were smokers also) that CO_2_ laser was effective at 6–8 W, in pulsed mode of 0.2 secs, and 4 mm spot size in removing melanin pigmentation. They however reported a reappearance of the pigmentation after 3 weeks in the dogs and in a majority of patients (4 out of 7) after 24 months. Almost all the patients had pain, though only one patient had persistent inflammation in the healing period [66]. These findings also concur with those of Esen et al. (2004) [67], in which a super pulsed CO_2_ laser at 10 watts, 0.8 mm spot size, in pulsed mode of 10 milliseconds at a frequency of 20 Hz, was used for gingival depigmentation in 10 patients and 2 patients had a relapse in the 12th and 16th month, respectively.

Simsek Kaya et al. (2012) [19] showed through their randomized prospective study that while the Er:YAG laser at 1 W, using a 1 mm sapphire tip in non-contact mode was equally as effective in depigmentation as the 810 nm diode laser at 1 W, 300 µm fiber tip in contact mode, the patients were more comfortable with the Er:YAG laser than with the diode. Nevertheless, they reported that follow-up over a 24 month period did not show any repigmentation in patients of both the diode laser and the Er:YAG laser group

Gholami et al. (2018) in a randomized clinical trial evaluated and compared the effectiveness of depigmentation done by scalpel surgical stripping as against using the Er,Cr:YSGG laser at 4.5 W and 2.5 W in a split-mouth approach. They evaluated pain using the VAS, while healing and bleeding was evaluated on an arbitrary scale. Reappearance of pigmentation was evaluated after 1 year according to the Hedin’s classification. They reported that most patients had lesser pain under both the Er,Cr:YSGG laser settings, and patient satisfaction was comparable between scalpel and both laser settings. Epithelial healing was more complete in the patients treated by both the laser settings compared to the scalpel group at 7 days, whilst relapse rates after 1 year were comparable between scalpel depigmentation and both groups of laser settings [16].

Nammour et al. (2020) compared the relapse rate after depigmentation (over 5 years) between three different laser wavelengths (Er:YAG, CO_2_, and diode laser 980 nm) in a randomized clinical trial on smokers and non-smokers. They used the Hedin’s Classification to evaluate the reappearance of gingival pigmentation and found that the relapse rate was faster in the smokers group, while the diode laser group had the most enduring results in both smokers (average, 29 months) and non-smokers (average, 39 months), Er:YAG laser group showed the earliest return of pigmentation (average, 7 months) in smokers and non-smokers (average, 9 months), whereas the CO_2_ laser group had an intermediate range of relapse between the three laser wavelengths (average, 24 months) and non-smokers (average, 28 months) [17].

Although healing of laser wounds is slower than healing of scalpel wounds, post-operatively, a sterile inflammatory reaction, hemostasis, little wound contraction, painless healing without any swelling, and precluding the use of antibiotics, analgesics or surgical dressings, and significantly, the patient’s freedom to enjoy normal food habits are advantages of laser-assisted depigmentation [68].

The reason why there is little or no relapse of pigmentation following laser-assisted depigmentation as compared to electrosurgery and scalpel is not clear. However, there are a few reports of repigmentation occurring over varied time periods. According to the migration theory by Land [69], active melanocytes from the adjacent pigmented tissues migrate to treated areas, causing a relapse, which is termed as melanocyte shift. For reasons as yet unexplained, this spontaneous phenomenon of melanocyte shift after the depigmentation procedure does not occur with laser depigmentation. This is probably because the laser effects complete removal of the epithelium up to the basement membrane [2,70].

## 5. Conclusions

Depigmentation is always an elective cosmetic procedure that is born out of the patient’s desire to achieve a beautiful smile. Variables such as lip line in relation to the teeth; dental components of the smile (teeth size, shape, and position); and the gingival constituents of color, contour, and texture of the gingiva all harmonize to give an aesthetic result. Precise and balanced assessments of these factors add up to give a balanced clinical judgment and diagnosis before performing a cosmetic procedure such as depigmentation. Similarly, Meleti and Vescovi (2008) [71] suggested a flow chart that would help in diagnosis by differentiating between pigmented lesions (malignant or benign). Furthermore, the use of clinical aids such as gingiva shade guides (IPS, IVOCLAR, VIVADENT or Lucitone 199, DENTSPLY, Trubyte) would help in pre-operative and definitive post-operative assessments and even in long term follow-up, especially from a legal and defining standpoint [72]. A painless and effective treatment modality restores not only the patient’s confidence but also contributes as a practice magnifier, in that, the patient would have the conviction and confidence to commit to further dental treatments.

Most of the articles discerned from the literature are case reports and are anecdotal in nature, controlled-randomized clinical trials and scientifically designed studies with adequate case numbers are lacking. Such studies with scientific and standardized protocols of evaluation are yet to provide a definitive take on comparative assessments between different techniques of depigmentation, the pain experienced by the patient, ease of healing, and relapse rates.

## Figures and Tables

**Table 1:** Dummett–Gupta Oral Pigmentation Index [2].

Dummett-Gupta Oral Pigmentation Index (DOPI)	
Score	Scale of Pigmentation
0	1. Pink–no pigmentation
1	2. Light Brown–mild pigmentation
2	3. Mixed Pink and Brown or Medium Brown
3	4. Deep Brown–Blackish Brown

**Table 2:** Hedin’s classification [9].

Hedin’s Classification	
Score	Scale of Pigmentation
Degree 1	Isolated—only 1 or 2 pigmented interdental papillae
Degree 2	Numerous pigmented interdental papillae
Degree 3	Short continuous ribbons
Degree 4	Long continuous ribbon

**Table 3:** Classification of gingival pigmentation by score 0–10 against pathology/causative factors. Source: [10].

Pigmented Lesions Index	Proposed Gingival Melanin Pigmentation
Score 0	Coral pink-colored gingiva, no gingival pigmentation, and/or pigmented lesions
Score 1	Mild, solitary/diffuse, gingival melanin pigmentation involving anterior gingiva, with or without the involvement of posterior gingiva
Score 2	Moderate to severe, solitary or diffuse, gingival melanin pigmentation involving anterior gingiva with or without the involvement of posterior gingiva
Score 3	Gingival melanin pigmentation only in posterior gingiva
Score 4	Tobacco-associated pigmentation: smoker’s melanosis, chewing tobacco
Score 5	Gingival pigmentation due to exogenous pigments: amalgam tattoos arsenic, bismuth, hewing betel nut, cultural gingival tattooing, drinks, food colors, lead-Burtonian line, mercury, silver, topical medications, idiopathic, etc.
Score 6	Gingival pigmentation due to other endogenous pigments: bilirubin, blood breakdown products, ecchymosis, hemochromatosis, hemosiderin, petechiae, etc.
Score 7	Drug-associated gingival pigmentation: antimalarial drugs, minocycline, oral contraceptives, etc.
Score 8	Gingival pigmentation associated with other causes: Addison’s disease, Albright’s syndrome, basilar melanosis with incontinence, hereditary hemorrhagic telangiectasia, HIV patients, lichen planus, neurofibromatosis, Peutz–Jeghers syndrome, pyogenic granuloma/granulomatous epulis, etc.
Score 9	Pigmented benign lesions: hemangioma, melanocytic nevus, pigmented macule
Score 10	Pigmented malignant lesions: angiosarcoma, Kaposi’s sarcoma, malignant melanoma

**Table 4:** Analysis of data table and systematic review data.

Citation [ref]	Type of Study/Number of Samples/Pocket Depth	Test/Control Groups	Laser Used	Scoring Used	Follow-up	Outcome
Basha et al. 2015 [14]	Clinical trial 20 patients 40 sites. Both sexes, 18–38 years Randomized, SB, comparative, split-mouth, clinical trial	Gr (i) Surgical stripping Gr (ii) laser	Nd:YAG 1064 nm Av Power 3.0 W 30 mj/pp 100 Hz Contact mode	Dummett Oral Pigmentation Index (DOPI) for intensity of pigmentation, Hedin Melanin Index for extent of pigmented area. VAS pain	6 months	Nd:YAG laser can be used as an alternative technique for gingival depigmentation No statistical significance for all results. Time with laser significantly less (*p* = 0.05)
Chandra et al. 2020 [15]	Split-mouth RCT. DB. 20 patients	Gr (i) Surgical stripping Gr (ii) laser	Diode 810 nm 1.5–2.0 W CW. Contact mode	Plaque and gingival index, bleeding, pain perception, wound healing, recurrence, and intensity of repigmentation (DOPI) were evaluated	No recurrence at 9 months were followed even up to 3 years.	Surgical scalpel technique remained as the “gold standard” procedure for treatment. Bleeding significantly less for laser (*p* = 0.05). No significant difference otherwise.
Gholami 2018 [16]	RCT DB 22 patients 66 sites	Gr (i) Laser 4.5 W Gr (ii) 2.5 W Gr (iii) Blade	Er,Cr:YSGG 2780 nm G. (i) 4.5 W, 50 Hz, 60 μs pulse, air, water G. (ii) 2.5 W, 50 Hz, 700 μs pulse, air, water	Bleeding, pain perception, wound healing, recurrence, and intensity of repigmentation (DOPI) were evaluated	1 month 12 months	Patient satisfaction/bleeding significantly better with laser (*p* = 0.05). Healing not statistically significant.
Nammour 2020 [17]	RC Study DB 72 patients	6 groups – 3 smokers/3 non-smokers	Diode 980 nm 1.5 W 50 Hz, fiber diameter 0.3 mm F = 21.22 J/cm^2^. Contact mode Er:YAG 2940 nm 5.0 W/20 Hz, 250 mJ, F = 39.31 J/cm^2.^ Non-contact mode CO_2_ 10,600 nm. 1.5 W SP 40 Hz, beam dia 0.3 mm, F = 17 J/cm^2^. Non-contact mode	Hedin Melanin Index (HMI) was used	2 weeks to 60 months All measured values within significance (*p* = 0.05).	Diode laser provides the longest-term stability in treatment. Smoking negatively affects the longevity of GD. Er laser gives the shortest time before the reappearance of gingival pigmentation.
Ribeiro 2014 [18]	Split-mouth RCT. DB. 11 patients	Gr. (i) Laser Gr. (ii) scalpel Random blinded determined operating side	Nd:YAG 6 W, 60 mJ/pulse, 100 Hz Contact mode	VAS Digitized photograph pixel count.	7, 15 days, 3, 6 months Significant differences in more pain, better patient acceptance (*p* = 0.05).	Nd:YAG laser has advantages in terms of less discomfort/ pain during the post-therapy period and a reduction of treatment chair time.
Simsek Kaya 2012 [19]	Randomized prospective study 20 patients 13 F, 7 M	Gr. (i) Diode 810 nm Gr. (ii) Er:YAG	Diode 810 nm 1.0 W CW Contact mode Er:YAG 2940 nm Av. Power 1.0 W Non-contact mode	Melzack’s McGill Pain Questionnaire Digitized photograph pixel count	Average pain scores: diode 1.5; Er:YAG 1.0 FU 7 days, 10–14 months.	Diode and Er:YAG lasers administered at 1 W both result in satisfactory depigmentation of GMP (*p* = 0.05) Diode surgically Quicker than Er:YAG (*p* = 0.05).

VAS: Visual Analog Scale.
